# Comparison of human glomerulus proteomic profiles obtained from low quantities of samples by different mass spectrometry with the comprehensive database

**DOI:** 10.1186/1477-5956-9-47

**Published:** 2011-08-10

**Authors:** Ying Zhang, Yutaka Yoshida, Bo Xu, Sameh Magdeldin, Hidehiko Fujinaka, Zan Liu, Masahito Miyamoto, Eishin Yaoita, Tadashi Yamamoto

**Affiliations:** 1 Department of Structural Pathology, Institute of Nephrology, Graduate School of Medical and Dental Sciences, Niigata University, Niigata, Japan; 2 Department of Physiology, Faculty of Veterinary Medicine, Suez Canal University, Ismailia, Egypt; 3 Institute of clinical Research, Niigata National Hospital, Kashiwazaki, Japan; 4 Division of Urology, Department of Regenerative and Transplant Medicine, Niigata Graduate School of Medical and Dental Sciences, Niigata, Japan; 5 Division of Nephrology and Hypertension, Department of Internal Medicine, St. Marianna University School of Medicine, Kawasaki, Japan

**Keywords:** human glomerulus, high-confident proteomics, microproteomics

## Abstract

**Background:**

We have previously constructed an in-depth human glomerulus proteome database from a large amount of sample for understanding renal disease pathogenesis and aiding the biomarker exploration. However, it is usually a challenge for clinical research to get enough tissues for large-scale proteomic characterization. Therefore, in this study, we focused on high-confidence proteomics analysis on small amounts of human glomeruli comparable to those obtained from biopsies using different mass spectrometers and compared these results to the comprehensive database.

**Results:**

One microgram of human glomerular protein digest was analyzed each on five LC- combined mass spectrometers (LIT-TOF, LTQ-Orbitrap, Q-TOF, LIT and MALDI-TOF/TOF) yielding 139, 185, 94, 255 and 108 proteins respectively identified with strict criteria to ensure high confidence (> 99%) and low false discovery rate (FDR) (< 1%). An integrated profile of 332 distinct glomerular proteins was subsequently generated without discerned bias due to protein physicochemical properties (pI and MW), of which around 60% were detected commonly by more than two LC-MS/MS platforms. Comparative analysis with the comprehensive database demonstrated 14 proteins uniquely identified in this study and more than 70% of identified proteins in small datasets were concentrated to the top abundant 500 in the comprehensive database which consists of 2775 non-redundant proteins.

**Conclusion:**

This study showed representative human glomerulus proteomic profiles obtained from biopsies through analysis of comparable amounts of samples by different mass spectrometry. Our results implicated that high abundant proteins are more likely to be reproducibly identified in multiple mass spectrometers runs and different mass spectrometers. Furthermore, many podocyte essential proteins such as nephrin, podocin, podocalyxin and synaptopodin were also identified from the small samples in this study. Bioinformatic enrichment analysis results extended our understanding of the major glomerular proteins about their subcellular distributions and functions. The present study indicated that the proteins localized in certain cellular compartments, such as actin cytoskeleton, mitochondrial matrix, cell surface, basolateral plasma membrane, contractile fiber, proteinaceous extracellular matrix and adherens junction, represent high abundant glomerular proteins and these subcellular structures are also highly significantly over-represented in the glomerulus compared to the whole human background.

## Introduction

The kidney glomerulus not only plays a pivotal role in plasma ultrafiltration but also is the locus of kidney diseases which frequently progress to irreversible chronic renal failure. We have previously performed large-scale proteomic analysis of human glomerulus and constructed an in-depth database (http://www.hkupp.org) for understanding the pathophysiology of renal diseases [1]. With the dramatic improvement of mass spectrometry (MS) instrumentation in last the years, proteomics research began moving from a large-scale to a micro mode for clinical applications due to the lack of substantial samples [2]. In this study, we aimed to analyze human glomerular proteins with low quantities which are comparable to those obtained from biopsies using different mass spectrometers and compare these results with the comprehensive database.

It has become the consensus that MS-based proteomics research would be difficult to deliver exactly same protein identification results for a complex sample while using different MS instruments and liquid chromatography (LC) separation methods. In addition, it was also surprisingly found that data interpretation strategy may be the most essential factor to decide whether the identification result could be correctly reported or not after a HUPO (Human Proteome Project) test sample study [3,4]. In this study, we analyzed one microgram of in-solution digested human glomerular proteins each on five mass spectrometers including LIT-TOF (linear ion-trap quadrupole- time of flight), LTQ-Orbitrap, Q-TOF (quadrupole-time of flight), LIT and MALDI-TOF/TOF (matrix-assisted laser desorption/ionization-time of flight/time of flight), and examined how different the identification results would be while using a same protein interpretation strategy. To deliver reliable protein identification list, we took stringent control of the confidence of each assigned peptide and the false discovery rate (FDR).

## Materials and methods

### Normal human glomerulus

This study was approved by the Ethics Committees of Niigata University Faculty of Medicine and conducted in accordance with their ethical principles. The kidney tissue was obtained from a 49-year-old male patient with his informed consent, who underwent nephrectomy due to renal cell carcinoma. The patient was normal in clinical examinations and did not receive any chemotherapy and radiotherapy. Pieces of cortex with normal appearance were excised and subjected to histological study and immunofluorescence chemistry to guarantee no pathological manifestations under light microscopy and no immunoglobulin (IgA, IgG, and IgM) and complement C3 deposits as described previously [1]. Glomeruli used for proteomics research were isolated by the standard sieving method with the purity over 95% as examined under a phase-contrast microscope as described in previous studies [5,6].

### In-solution trypsin digestion

In-solution trypsin digestion was carried out according to Mawuenyega et al [7]. Briefly, isolated human glomeruli were dissolved in the buffer containing 7 M guanidine-HCl, 0.5 M Tris, pH 8.5, and 10 mM EDTA-Na by ultrasonication and incubation at 37°C for 30 min with gentle agitation. The sample was DTT (dithiothreitol) reduced, iodoacetamide alkylated and then precipitated with methanol and chloroform. The precipitate was solubilized in the buffer containing 6 M urea, 0.1 M Tris-HCl, pH8.8 and subjected to the protein assay, the modified Lowry method. The dissolved sample was diluted with 0.1 M Tris-HCl, pH8.8 to the final urea concentration of 1.0 M. Trypsin (proteomics grade, Sigma, U.S.A) was then added at enzyme/substrate weight ratio of 1:50, and incubated at 37°C for 16 h. The sample was acidified with TFA (trifluoroacetic acid) to pH 2-3, and stored at -80°C until proteomics analysis.

### Mass spectrometry analysis

One microgram of trypsin-digested sample each was measured on five MS instruments including LIT-TOF, LTQ-Orbitrap, Q-TOF, LIT and MALDI-TOF/TOF. The HPLC (high-performance liquid chromatography) separation methods were elastically controlled according to instrumental features and the actual working state in order to acquire satisfying MS raw data (Table 1 for detailed MS and LC settings).

**Table 1:** Experimental settings of mass spectrometry analyses

Instrument _a)_	LIT-TOF	LTQ-Orbitrap	Q-TOF	LIT	MALDI-TOF/TOF
**LC settings:**					
Separation column	MonoCap 75 μm × 150 mm, C18, monolith	Michrom 75 μm × 50 mm, C18, 3 μm	Zorbax 75 μm × 150 mm, C18, 5 μm	MonoCap 50 μm × 250 mm, C18, monolith	KYA 100 μm × 150 mm, C18, 5 μm
Flow rate (nL/min)	200	500	300	200	300
Mobile phase gradient	2%B (0 min) → 40%B (100 min) → 100%B (100.1 min) → 100%B (120 min) → 2%B (120.1 min) → 2%B (140 min)	5%B (0 min) → 45%B (80 min) → 95%B (81 min) → 95%B (89 min) → 5%B (90 min) → 5%B (120 min)	3%B (0 min) → 40%B (50 min) → 100%B (55 min) → 100%B (65 min) → 3%B (66 min) → 3%B (80 min)	0%B (0 min) → 50%B (140 min) → 50%B (155 min) → 100%B (156 min) → 100%B (170 min)	0%B (0 min) → 50%B (60 min) → 100%B (80 min) → 100%B (90 min)
Data acquisition time (min)	120	90	65	170	90
**MS settings:**					
Instrument	Hitachi NanoFrontier LD	Thermo LTQ-Orbitrap Discovery	Agilent 6510 Q-TOF	GE& Thermo MDLC-LTQ	ABI 4800 MALDI-TOF/TOF
MS scan	TOF	Orbitrap	TOF	LIT	TOF
MS/MS scan	TOF	LTQ	TOF	LIT	TOF
MS/MS spectrum acquisition	IBA mode **_b)_**	IBA mode	IBA mode	IBA mode	IBA mode

**^a)^**Instrument: LIT, linear iontrap; Q, quadrupole; MALDI, matrix-assisted laser desorption/ionization; TOF, time of flight.

**^b)^**IBA mode: information-based acquisition mode. MS/MS spectra acquired from the first run are excluded and would not be detected in the second run. Instead, the MS/MS spectra with lower intensity than the excluded ones would be obtained in the second run.

The peptide sample was analyzed twice by each mass spectrometer (0.5 μg × 2). The three most intense precursor ions were selected and fragmented to produce MS/MS spectra for each MS scan. IBA (information-based acquisition) mode was the principal MS/MS data acquisition strategy used in the two analysis runs, by which the MS/MS spectra acquired from the first run would be recorded and not be detected in the second run. Instead, the MS/MS spectra with lower intensity than the excluded ones would be obtained in the second run. Finally, MS raw data of twice analyses were merged through MASCOT Daemon under the instruction manual and then subjected to MASCOT MS/MS ion search.

### Data processing

The acquired MS/MS spectra were searched with MASCOT (Ver. 2.2) algorithm against IPI human database (Ver. 3.70, 87,069 sequences). Peptide and MS/MS tolerance for each mass spectrometer were set as: LTQ-TOF, ± 0.1 Da, ± 0.1 Da; LTQ-Orbitrap, ± 20 ppm, ± 0.5 Da; Q-TOF, ± 0.2 Da, ± 0.2 Da; LTQ, ± 2.0 Da, ± 0.8 Da; MALDI-TOF/TOF, ± 0.2 Da, ± 0.3 Da. Carbamidomethylation on cysteine was set as fixed modification. Oxidation on methionine were set as variable modifications. One trypsin missed cleavage was allowed. For enhancing the confidence of identified proteins and reducing the data redundancy, we took four strict format controls during MASCOT search as follows.

1. MudPIT (multidimensional protein identification technology) protein scoring method was used for removing junk protein hits, which have high protein scores but no high scoring peptide matches.

2. FDR (false discovery rate) was controlled lower than 1% by adjusting the significance threshold p-value.

3. The option *Expect cut-off value *was set as the same as the significance threshold *p*-value to ensure that only high confident peptides with their ions score higher than the identity threshold were selected for protein identification.

4. The option *Require bold red *was checked *on *to remove the redundant homologous proteins and to ensure at least one unique peptide assigned to the hit protein.

### Bioinformatics analysis

Identified proteins were categorized by protein class through PANTHER (Protein ANalysis THrough Evolutionary Relationships) analytic tool (Ver. 7.0) [8,9]. The protein distribution in subcellular locations was analyzed using GO CC (Gene Ontology cellular component), which is a controlled vocabulary on the parts of a cell and its extracellular environment [10], via DAVID tool (Database for Annotation and Integrated Discovery) (Ver. 6.7), hosted by NIAID/NIH (National Institute of Allergy and Infectious Diseases/National Institutes of Health) aiming at functional annotation and enrichment analysis [11,12].

Functional enrichment analyses of cellular component (CC), molecular function (MF), biological process (BP), and KEGG (Kyoto Encyclopedia of Genes and Genomes) pathway were performed via DAVID analytic tool. In the enrichment analysis, a test dataset (which is a human glomerulus dataset obtained in this study or the comprehensive database) was compared to the reference DAVID human knowledgebase, consisting of 54,981 gene IDs parsed from around 45,000 Entrez gene IDs and 35,000 Ensembl gene IDs. GO FAT, which is a collection of child GO categories with the broadest ones filtered out, was utilized as the ontology file in order to define the specificity of a given term. Modified Fisher Exact test was used for statistics analysis. The significantly enriched GO categories (p-value < 0.001) and KEGG pathways (p-value < 0.05) were presented.

## Results and Discussion

### Protein identification

In this study, we performed LC-MS/MS analysis by using information based acquisition (IBA) technique in order to obtain more precursor ions with lower intensity for MS/MS fragmentation among separate MS runs [13]. We also conducted the preliminary experiment of four IBA runs for one sample analysis in order to assess the efficiency of every IBA run on both peptide and protein identification (see Additional file 1). As a result, the increase of non-redundant peptide and protein identification became very small after the third run, and the cumulative identification in the first two runs covered almost 80% of total yield of all the four runs. Although the more runs would be more preferable for protein yield, we measured the sample twice in each IBA mode analysis due to the limited amount of protein digest. Trypsin digests from normal human glomerular proteins were analyzed on five mass spectrometers and the obtained MS/MS spectral raw data was processed by MASCOT search engine against IPI human database. For MS/MS ion search, MASCOT has its advantage of calculating the significance threshold (identity threshold) of ions score for every assigned peptide and only those peptides above their identity threshold indicate high reliability. Therefore, we considered not only the number of distinct peptides assigned to a given protein but also the quality of assigned peptides for assessing the confidence of each hit protein. Briefly, we adopted a protein identification strategy (see **Methods and Materials**) focused on low FDR, high peptide reliability and protein non-redundancy for improving the confidence of identified proteins. We confined those proteins meeting the following criteria as high confident ones: those have at least two distinct peptides with their ions scores greater than their identity thresholds and out of these two peptides at least one is unique to the matched protein.

**Figure 1:**
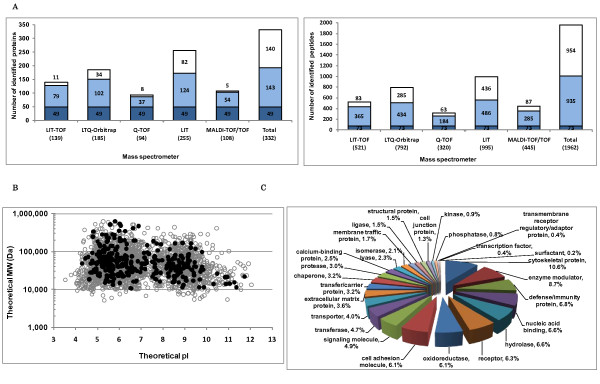
**Peptide and protein identification from low quantities of normal human glomeruli using five mass spectrometers**. **A**. Protein digests of human glomeruli were analyzed on five mass spectrometers including LIT-TOF, LTQ-Orbitrap, Q-TOF, LIT and MALDI-TOF/TOF, yielding 139, 185, 94, 255, 108 proteins respectively (significance p < 0.01, FDR < 1%), and 521, 792, 320, 995 and 445 peptides respectively (ions score > identity threshold). An integrated profile of 332 proteins was subsequently generated. Blue column represents the number of proteins identified by all the MS instruments, while light blue by at least two (2~4) and white uniquely by only one. **B**. Protein distribution patterns for the integrated profile and the comprehensive one were plotted due to their physicochemical properties (pI and MW) and no discernible bias was observed. Gray circles, proteins in the comprehensive database; black spots, proteins in the integrated dataset. **C**. The total glomerular proteins identified in this study were categorized by protein class, ranking cytoskeletal proteins to the top with the largest proportion.

Mass spectrometry analyses of one microgram of human glomerular protein yielded 521, 792, 320, 995 and 445 distinct high-confident peptides which were separately matched to 139, 185, 94, 255 and 108 proteins by LIT-TOF, LTQ-Orbitrap, Q-TOF, LIT and MALDI-TOF/TOF MS instruments respectively (Figure 1A). An integrated profile of 332 non-redundant proteins was subsequently constructed, of which 49 proteins (15%) were identified by all 5 MS instruments and 192 proteins (60%) by at least two MS instruments. Among the identified proteins, 182 (55%) were identified as high confident proteins and 150 (45%) as low confident ones, which repeats the same confidence level of protein yield as the comprehensive database, and furthermore, among 150 low-confidence proteins, there were 43 that were detected by more than two MS instruments indicating their reproducibility in MS measurement. The detailed information for the total identified proteins is showed in Additional file 2 and the MS/MS data may be downloaded from Tranche of ProteomeCommons.org. From our previous data on human glomeruli, the amount of extracted proteins from one human glomerulus, by using SDS containing lysis buffer, is about 0.6 μg (data not shown). Therefore, we consider that the small datasets obtained in this study provide the representative microproteomic profiles of human glomeruli from biopsies.

We divided the five LC-MS/MS systems into two groups: one consists of three LIT specific platforms and the other one consists of MALDI-TOF/TOF and Q-TOF, and examined the overlapping of peptide and protein identifications between two groups (see Additional file 3). As a result, 91% of proteins and 74% of peptides detected in the group containing MALDI-TOF/TOF and Q-TOF are covered by those identified in the LIT specific platforms implicating similarity in detecting precursor ions by different LC-MS/MS platforms.

Although the protein numbers of the small datasets are relatively smaller than that of the comprehensive data, these numbers are relatively satisfactory when considering the low quantity of consumed tissue. We also examined the proteins identified in this study. From the integrated data, we found quite a number of proteins which play essential roles in podocytes including nephrin, podocin, podocalyxin, synaptopodin, ZO-1, ezrin, integrins and ILK, of which nephrin, synaptopodin and integrin alpha-V were identified in only one dataset while the others in at least two. In addition, the dataset obtained from LIT platform covered all the proteins mentioned above, suggesting that a dataset of the protein yield more than 200 might be preferable for microproteomics research of human glomeruli.

We then examined the distribution patterns according to protein physicochemical properties (theoretical pI and molecular weight (MW)). Figure 1B showed the comparison of the integrated profile (332 proteins) with the comprehensive one (2775 proteins) and no discerned bias was found. Of the 332 proteins, around 60% (188) had theoretical pI values in the pH 5-9 range and MW between 20 kDa to 100 kDa, while a number of proteins with extreme pI or MW were also found. A total of 22 acidic proteins with a theoretical pI of < 5.0 and 10 basic proteins with a theoretical pI of > 10.0 were identified; 13 small proteins with theoretical MW < 15 kDa and 37 proteins with theoretical MW > 150 kDa were identified.

The total 332 proteins were categorized by protein class (Figure 1C), ranking cytoskeletal proteins to the top (10.6%), which is very similar to that (12%) obtained from the comprehensive database. In addition, enzyme modulator (8.7%), defense/immunity protein (6.8%), nucleic acid binding (6.6%) and hydrolase (6.6%) were also found as the major protein classes. On the other hand, a small number of proteins were classified to surfactant, transcription factor, transmembrane receptor regulatory/adaptor protein, phosphatase and kinase. Furthermore, we found 14 proteins uniquely identified in this study when compared with the comprehensive database and two out of them were identified with at least two distinct peptides. The majority of these proteins were intracellular organelle proteins, of which there were three cytoskeletal proteins including tropomodulin-2, which is poorly understood in kidney so far. We also found four integral membrane proteins including DPP4 (dipeptidyl peptidase 4) which has been examined in rat glomerulus as a tyrosine-phosphorylated protein in our previous study [14] and SLCO2A1 (solute carrier organic anion transporter family member 2A1) which was not reported in kidney before. The detailed information for the 14 proteins which are uniquely identified in this study is presented in Additional file 2.

### Comparison of protein subcellular distribution patterns of small datasets with that of the comprehensive database

**Figure 2:**
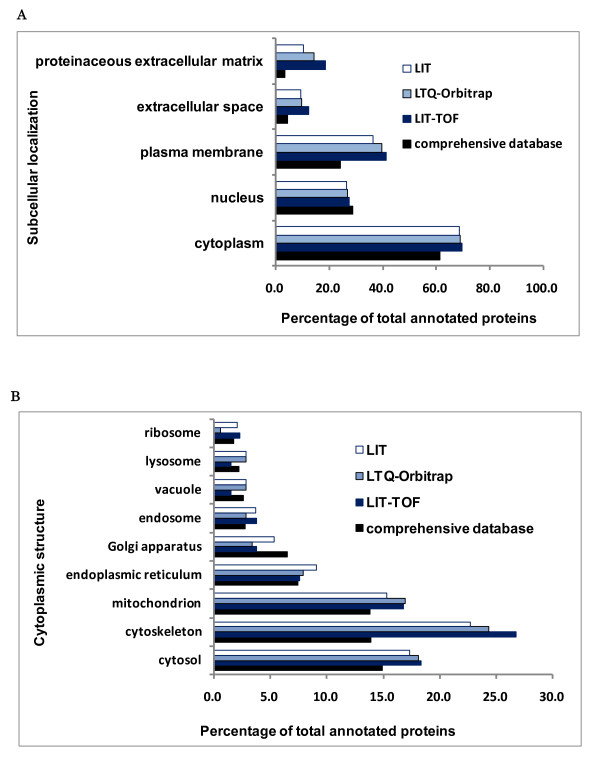
**Comparison of protein subcellular distribution patterns between three small datasets and the comprehensive database**. **A**. Presented was protein distribution patterns of five major subcellular components. **B**. Presented was protein distribution patterns of detailed cytoplasmic structures.

Using Gene Ontology cellular component (GO CC) vocabulary, proteins were parsed into major subcellular compartments including cytoplasm, plasma membrane, nucleus, extracellular space and proteinaceous extracellular matrix (Figure 2A) and detailed cytoplasmic structures (Figure 2B). We found that the cytoplasmic and nuclear proteins of small datasets obtained from LIT-TOF, LTQ-Orbitrap and LIT specific platforms have similar proportions to those of the comprehensive database. However, the proportions of plasma membrane proteins (around 40%) and extracellular proteins (extracellular space and proteinaceous extracellular matrix) (around 25%) of the small datasets are higher than the comprehensive database (around 24% and 7.5%), possibly due to the different amounts of samples used in this study (1 μg) and the previous large-scale analysis (~ 2 mg) resulting in a number of low-abundance proteins missed in small datasets. In addition, cytoskeleton, cytosol and mitochondrion were found as top three cytoplasmic structures with largest proportions of annotated proteins.

### Comparison of relative abundance of common proteins identified in the small datasets and the comprehensive database

**Figure 3:**
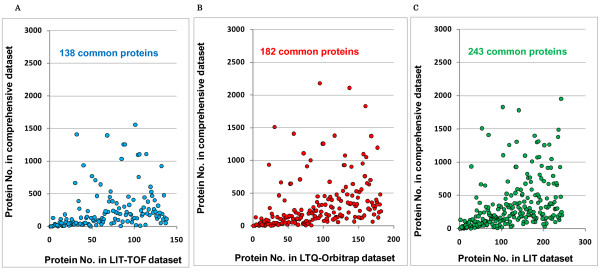
**Protein match and distribution patterns of small datasets to the comprehensive database**. The identified proteins in the small and comprehensive dataset were firstly ranked due to their spectra count (from highest to lowest) and then the graph for the commonly detected proteins was plotted according to their orders. **A**. LIT-TOF dataset versus the comprehensive database (138 common proteins). **B**. LTQ-Orbitrap dataset versus the comprehensive database (182 common proteins) **C**. LIT dataset versus the comprehensive database (243 common proteins).

We ordered proteins according to their spectral count (from highest to lowest) and then the common proteins between small datasets (LIT-TOF, LTQ-Orbitrap and LIT) and the comprehensive database were plotted due to their orders. As a result, 138 out of 139 proteins in LIT-TOF dataset, 182 out of 185 proteins in LTQ-Orbitrap dataset, and 243 out of 255 proteins in LIT dataset were commonly identified in the comprehensive database (Figure 3). We found that more than 70% of proteins in small datasets were concentrated to the top abundant 500 in the comprehensive database, with more than 90% to the top abundant 1000, suggesting that human glomerular proteins identified from small samples (1 μg) represent the part of proteins with high abundance in the comprehensive database.

### Functional enrichment analysis of GO annotation and KEGG pathway

The identified proteins in three small datasets from LIT specific platforms and the comprehensive database were categorized based on GO hierarchy vocabulary and KEGG pathway, and then compared to the complete human proteome for picking up the significantly over-represented GO categories and KEGG pathways.

#### GO cellular component

**Figure 4:**
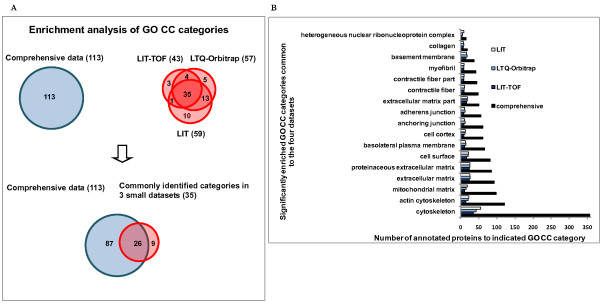
**Comparison of enrichment analysis results of GO CC (cellular component) among three small datasets and the comprehensive database**. **A**. The graph represents the number of annotated GO CC categories of each dataset which were found to be significantly enriched (p < 0.001) in the glomerulus. The overlapping among the four datasets is also shown. **B**. Data are presented as a bar chart of 17 over-represented subcellular structures except melanosome and its related categories which were commonly identified in the four datasets.

We compared the over-represented GO cellular component categories in the small datasets and the comprehensive database as shown in Figure 4. Twenty-six of enriched subcellular structures (p < 0.001), including *actin cytoskeleton*, *mitochondrial matrix, cell surface, basolateral plasma membrane, adherens junction, myofibril, basement membrane, heterogeneous nuclear ribonucleoprotein complex*, and so on, were commonly found in the four datasets which greatly extends our understanding of the subcellular distribution pattern of major glomerular proteins. Furthermore, these subcellular structures are also implicated as highly significantly enriched GO CC categories when compared with the whole human background.

In this study, cytoskeletal proteins occupy the highest proportion of total identified proteins of all datasets with high enrichment significance. Podocytes in the glomerulus are well known for their unique structure characterized by the interdigitating foot processes. The highly dynamic foot processes are rich in the actin-based cytoskeleton which plays vital roles not only for cellular organization integrity but also for podocyte signaling pathways [14-16]. Our results support these facts. A total of 34 proteins from the integrated dataset were identified as actin cytoskeleton proteins. Besides actins (ACTA1, ACTA2), myosins (MYL6B, MYO1B, MYO1C, MYH9, MYH10) and their associated proteins (CAPZA1, CAPZA2, CAPZB, ACTR2, ACTR3, ARPC1B), and other well known glomerular proteins (alpha-actinin-4 [17,18], synaptopodin [19], IQGAP [20], ezrin [21], vinculin [22], and ILK (integrin-linked protein kinase) [23]), we also found some proteins, such as gelsolin, SLC9A3R1 (Na+/H+ exchange regulatory cofactor NHE-RF1), and fascin with little report in glomerulus, and plastin-2, PDLIM5 (PDZ and LIM domain protein 5), ALDOA (fructose-bisphosphate aldolase A) with no report in kidney so far. Further study would be required for investigating these proteins in the kidney.

On the other hand, we were very surprised to find out that *melanosome *was also demonstrated as a significant over-represented subcellular location both in the small datasets (p < E-13) and in the comprehensive database (p < E-22). Melanosome is defined as a membrane-bounded cytoplasmic vesicle within which melanin pigments are synthesized and stored. Melanosome is usually produced in skin, hair and eye involving in the synthesis and dispersion of melanin to neighboring keratinocytes [24]. Although melanosome was found in malignant pigmented epithelioid tumor cells of kidney [25,26], there is no report for its existence in normal kidney. Furthermore, there are no melanosome-specific proteins, such as tyrosinase-related proteins (TYRP) and Pmel 17 (SILV) [27,28], examined in this study or the large-scale analysis. Therefore, *melanosome *and other 8 of melanosome-related categories were finally excluded from our list for the common over-represented subcellular structures in the glomerulus (Figure 4B). Why was *melanosome *detected as an over-represented cellular component? The possible reason is that melanosomes share diverse proteins with other subcellular compartments, such as plasma membrane and some organelles. For example, we identified a total of 27 melanosome proteins in this study, and in fact, they were also found in plasma membrane (10), endoplasmic reticulum (9), mitochondrion (6), Golgi apparatus (5), cytosol (5) and so on.

#### GO molecular function

**Figure 5:**
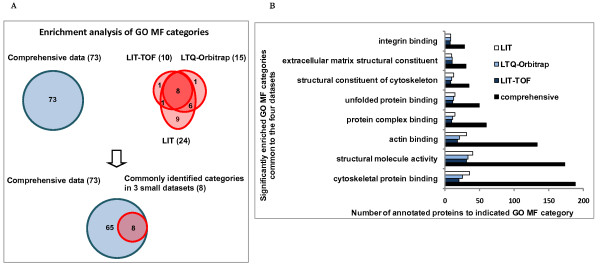
**Comparison of enrichment analysis results of GO MF (molecular function) among three small datasets and the comprehensive database**. **A**. The graph represents the number of annotated molecular functions of each dataset which were found to be significantly enriched (p < 0.001) in the glomerulus. The overlapping among the four datasets is also shown. **B**. Data are presented as a bar chart of 8 over-represented molecular functions which were commonly identified in the four datasets.

Enrichment analysis of GO molecular function indicated that eight categories were commonly detected in both small datasets and the comprehensive one with high enrichment significance (p < 0.001) compared with the human background (Figure 5). As expected, *cytoskeletal protein binding, structural molecule activity *and *actin binding *were ranked to the top, suggesting the prominent biological meaning of cytoskeleton in the glomerulus. In addition, other binding functions (*protein complex binding*, *unfolded protein binding *and *integrin binding*) and molecule actions contributing to the structural integrity (*structural constituent of cytoskeleton *and *extracellular matrix structural constituent*) were also detected as over-represented categories in human glomeruli.

#### GO biological process

**Figure 6:**
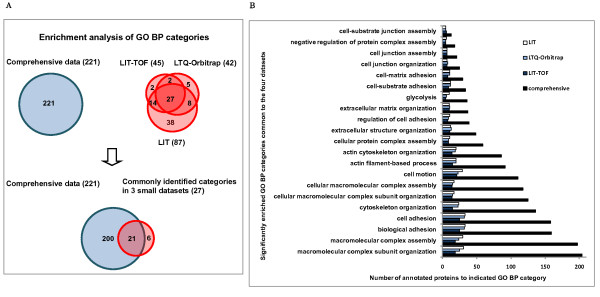
**Comparison of enrichment analysis results of GO BP (biological pathway) among the three small datasets and the comprehensive database**. **A**. The graph represents the number of annotated biological processes of each dataset which were found to be significantly enriched (p < 0.001) in the glomerulus. The overlapping among the four datasets is also shown. **B**. Data are presented as a bar chart of 21 over-represented biological processes which were commonly identified in the four datasets.

Enrichment analysis of GO biological process showed that 21 categories were commonly detected in both small datasets and the comprehensive one with high enrichment significance (p < 0.001) compared with the human proteome background (Figure 6). These biological pathways include not only the cytoskeleton-related processes (*actin cytoskeleton organization *and *actin filament-based process*), but also extracellular structure-, cellular macromolecule- related processes (*extracellular matrix organization and cellular macromolecular complex assembly*), as well as *cell adhesion *and *cell motion*.

#### KEGG pathway

**Figure 7:**
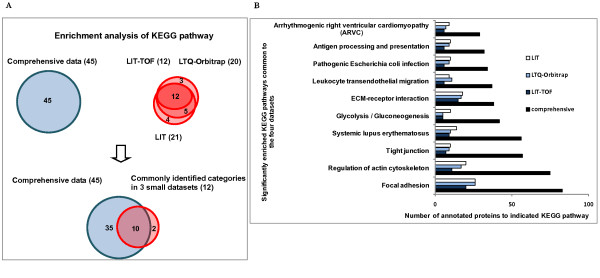
**Comparison of enrichment analysis results of KEGG pathway among the three small datasets and the comprehensive database**. **A**. The graph represents the number of annotated pathways of each dataset which were found to be significantly enriched (p < 0.05) in the glomerulus. The overlapping among the four datasets is also shown. **B**. Data are presented as a bar chart of 10 over-represented pathways which were commonly identified in the four datasets.

Significant over-represented KEGG pathways (p < 0.05) in the small datasets and the comprehensive database were compared and those commonly identified were shown in Figure 7. Among the ten common over-represented pathways, three are involved in cell-cell and cell-matrix contacts including *tight junction*, *focal adhesion *and *ECM-receptor interaction*, suggesting the biological importance of cell adhesion in human glomeruli. In addition, *regulation of actin cytoskeleton *was also found with high enrichment significance, implicating cooperative biological activities between actin cytoskeletal proteins and cell adhesion structures in the glomerulus. Glucose metabolism in the glomerulus might be quite active *as glycolysis/gluconeogenesis *was also identified as an over-represented pathway. On the other hand, KEGG pathway analysis also detected some over-represented disease pathways including *systemic lupus erythematosus*, *arrhythmogenic right ventricular cardiomyopathy *(ARVC) and *pathogenic Escherichia coli infection*, and two immune pathways including *antigen processing and presentation*, and *leukocyte transendothelial migration*, inferring that glomerular proteins might participate in diverse cellular pathological processes.

## Conclusions

In this study, we analyzed low quantities of human glomerular proteins by different LC-MS/MS systems, exhibiting representative human glomerulus proteomic profiles obtained from biopsies through analysis of comparable amounts of samples by mass spectrometry. Comparison of small datasets constructed in this study with the comprehensive database indicated that proteins identified in microproteomics analysis represent the part of proteins with high abundance in the comprehensive database without any discernible protein physicochemical bias. Furthermore, enrichment analysis of GO annotation and KEGG pathway presented the structural and functional distribution patterns of major glomerular proteins which were significantly enriched in human glomeruli when compared to the whole human background.

## List of abbreviations

**ARVC**: right ventricular cardiomyopathy; **BP**: biological process; **CC: **cellular component; **DAVID**: Database for Annotation and Integrated Discovery; **FDR**: false discovery rate; **GO**: gene ontology; **HUPO**: Human Proteome Project; **KEGG**: Kyoto Encyclopedia of Genes and Genomes; **LC-MS/MS**: liquid chromatography - tandem mass spectrometry; **LIT-TOF**: linear ion-trap - time of flight; **MALDI-TOF/TOF**: matrix-assisted laser desorption/ionization; **MF**: molecular function; **MW**: molecular weight; **NIAID/NIH**: National Institute of Allergy and Infectious Diseases/National Institutes of Health; **PANTHER**: Protein ANalysis THrough Evolutionary Relationships; **Q-TOF**: quadrupole - time of flight.

## Competing interests

The authors declare that they have no competing interests.

## Authors' contributions

YZ conceived the study, conducted MS raw data processing and bioinformatics analysis and drafted the manuscript. YY performed in-solution digestion and participated in mass spectrometry analysis. BX participated in mass spectrometry analysis and helped draft manuscript. SM participated in MS raw data processing. HF participated in bioinformatics analysis. ZL participated in MS raw data analysis. MM contributed to mass spectrometry analysis and discussion on bioinformatics analysis result. EY contributed to experiments for sample preparation and discussion on protein identification results. TY contributed to overall design of this study and strict revision of the manuscript for important intellectual content. All authors read and approved the final manuscript.

## Supplementary Material

Additional file 1 **Cumulative protein and peptide identification results of each run (from 1st to 4th) in the IBA mode by using LIT-TOF MS instrument**. Click here for file

Additional file 2 **A total of 332 human glomerular proteins identified by five mass spectrometers**. Click here for file

Additional file 3 **Comparison of peptide and protein identification results between the linear ion-trap conjugated LC-MS/MS group (LIT-TOF, LTQ-Orbitrap and LIT) and the other group (Q-TOF and MALDI-TOF/TOF)**. Click here for file
