# Supplementary material for: Comparison of human glomerulus proteomic profiles obtained from low quantities of samples by different mass spectrometry with the comprehensive database
Source: Proteome Sci. 2011 Aug 10;9:47. doi: 10.1186/1477-5956-9-47 (PMC3175441; doi:10.1186/1477-5956-9-47)
Supplement: Additional file 1 — Cumulative protein and peptide identification results of each run (from 1st to 4th) in the IBA mode by using LIT-TOF MS instrument. [file 1477-5956-9-47-S1.DOC]

**Additional file 1**

**A B**

**Additional file 1.** Cumulative, non-redundant peptide **(A)** and protein **(B)** identification results of each run by IBA (information-based acquisition) mode using LIT-TOF MS instrument. A total of 0.5 μg of glomerular protein digest each was measured four times by IBA mode. The blue columns indicate the number of cumulative peptides or proteins identified in the former runs and the red columns represent the new identifications in the current run. The number of MS/MS measurements of each run is 954, 960, 1050 and 982 respectively. As the figures shown, from the 4th run, both peptide and protein identifications increase more slowly than the former runs.
