# Supplementary material for: Comparison of human glomerulus proteomic profiles obtained from low quantities of samples by different mass spectrometry with the comprehensive database
Source: Proteome Sci. 2011 Aug 10;9:47. doi: 10.1186/1477-5956-9-47 (PMC3175441; doi:10.1186/1477-5956-9-47)
Supplement: Additional file 3 — Comparison of peptide and protein identification results between the linear ion-trap conjugated LC-MS/MS group (LIT-TOF, LTQ-Orbitrap and LIT) and the other group (Q-TOF and MALDI-TOF/TOF). [file 1477-5956-9-47-S3.DOC]

**Additional file 3**

**A B**

**Comparison of the identified peptides among the 5 datasets**

**Total identified peptides in LIT-TOF, LTQ-Orbitrap and LIT (1529)**

**Total identified peptides in Q-TOF and MALDI-TOF/TOF (622)**

**Comparison of the identified proteins among the 5 datasets**

**LIT-TOF (139)**

**LTQ-Orbitrap (185)**

**LIT (255)**

**Q-TOF (94)**

**MALDI-TOF/TOF (108)**

**LIT-TOF (521)**

**LTQ-Orbitrap (792)**

**Q-TOF (320)**

**MALDI-TOF/TOF (445)**

**LIT (995)**

**Total identified proteins in LIT-TOF, LTQ-Orbitrap and LIT (319)**

**Total identified peptides in Q-TOF and MALDI-TOF/TOF (141)**

**Additional file 3.** Comparison of peptide **(A)** and protein **(B)** identification results between the linear ion-trap conjugated LC-MS/MS group (LIT-TOF, LTQ-Orbitrap and LIT) and the other group (Q-TOF and MALDI-TOF/TOF).
